# Long-term outcomes of more than a decade treating patients with stereotactic body radiation therapy for hepatocellular carcinoma

**DOI:** 10.1016/j.ctro.2024.100878

**Published:** 2024-10-18

**Authors:** Wilhelm den Toom, Eva M. Negenman, Francois E.J.A. Willemssen, Erik van Werkhoven, Robert J. Porte, Roeland F. de Wilde, Dave Sprengers, Imogeen E. Antonisse, Ben J.M. Heijmen, Alejandra Méndez Romero

**Affiliations:** a Department of Radiotherapy, Erasmus MC Cancer Institute, Erasmus University Medical Center, Rotterdam, The Netherlands; b Departments of Radiology and Nuclear Medicine, Erasmus University Medical Center, Rotterdam, The Netherlands; c Erasmus MC Transplant Institute, Department of Surgery, Division of HPB and Transplant Surgery, Erasmus University Medical Center, Rotterdam, The Netherlands; d Department of Gastroenterology & Hepatology, Erasmus MC Transplant Institute, Erasmus University Medical Center, Rotterdam, The Netherlands

**Keywords:** SBRT, Long term outcomes, Hepatocellular carcinoma

## Abstract

**Purpose/Objective(s):**

To evaluate if stereotactic body radiotherapy (SBRT) for hepatocellular carcinoma (HCC) has a durable effect on tumor control and can be delivered safely.

**Materials/Methods:**

Patients included in this retrospective study have been treated at our institution from January 2008 to December 2022. Eligibility criteria were diagnosis of HCC, BCLC stage 0-A-B, non-cirrhotic liver or liver with cirrhosis Child-Pugh class A, and a maximum of three lesions with a cumulative diameter of ≤ 6 cm. Patients with relapses after surgery, thermal ablation or TACE or patients awaiting transplant were also candidates for SBRT. SBRT was delivered in 6 fractions of 8 or 9 Gy. The primary endpoint was local (target) control (LC). Secondary endpoints were time to progression (TTP), overall survival (OS), response rate (RR) and toxicity.

**Results:**

A total of 52 patients received SBRT at our institution and 51 were included in this study. One patient objected and was excluded. Median follow-up was 2.1 years for LC and 2.3 years for OS. Median tumor size was 26 mm. LC rates at 1, 2, and 5 years were 100 %, 95 % and 95 % respectively. Median TTP was 45.6 months. Median OS was 7.1 years. RR was 96 %. No patients in this study have experienced SBRT related CTC AE grade ≥ 3 toxicity.

**Conclusion:**

SBRT resulted in excellent long-term local control rates and absence of severe toxicity in a group of HCC patients. The reported outcomes compare favorably with other local therapies. SBRT should be considered as one of the available local treatment options for HCC.

## Highlights

• SBRT resulted in a 5-year LC of 95% in the treatment of primary liver cancer (HCC).
• No SBRT related ≥ grade 3 toxicity was reported.
• Treatment outcomes of SBRT appear favorable to alternative local ablative therapies.
• SBRT should be considered as a local treatment amongst the other ablative therapies.

## Introduction

Primary liver cancer, and predominantly hepatocellular carcinoma (HCC), is the sixth most common cancer and the third most leading cancer death worldwide (GLOBOCAN 2022) [1]. Major risk factors for HCC are chronic Hepatitis B or C virus (HBV or HCV) infection or liver cirrhosis. More than 90 % of patients diagnosed with HCC suffer from underlying liver disease [2].

The treatment of HCC depends on the stage of the disease at diagnosis. Surgical resection or liver transplantation are considered the first choice of treatment for patients with limited disease [3], [4], [5]. However, only 15–30 % of patients qualify for surgery and the availability of donor livers is limited [6]. For patients who are not eligible for surgery, locoregional therapies (LRT) such as radiofrequency ablation (RFA), microwave ablation (MWA), transarterial chemoembolization (TACE) or selective internal radiotherapy (SIRT) are alternative treatment options [7], [8]. Depending on patient and tumor characteristics, the intent of LRT treatment for HCC can be bridging or down staging towards liver transplantation, curation or delaying disease progression [9]. The most commonly used staging system for HCC in the western world is the Barcelona Clinic liver cancer (BCLC) system which assigns treatments and prognoses to different stages of the disease [10]. The expected survival of HCC patients is dependent on eligibility for surgery or transplantation, number and size of tumor(s), patient performance score and liver function and is predicted by the BCLC to range from > 5 years for early stage to 3 months for terminal stage liver cancer [4].

Radiation therapy was historically not considered as a treatment option for HCC due to the risk of radiation induced liver disease. However, technological advances have allowed for a more precise radiation dose delivery in the liver. Stereotactic body radiation therapy (SBRT) uses a limited number of fractions with a high degree of precision and a sharp dose fall-off, to deliver a high radiation dose to the tumor while limiting the radiation dose to the liver parenchyma [10].

In the last decade, several prospective and retrospective studies investigating SBRT for HCC have demonstrated promising results with 1- and 2-years local tumor control rates ranging from 87 % to 97 % with no or limited toxicity [11], [12], [13], [14], [15], [16]. The reported survival in these studies shows a large heterogeneity associated with variations in the characteristics of the patients included in them. There is still limited supporting evidence from randomized control trials, who often report on small and incomplete populations due to slow accrual [17], [18]. This provides a challenge to prove the full potential and the position of SBRT in the HCC treatment paradigm.

Despite the increasing evidence for the efficacy of SBRT in the treatment of HCC, its role is not yet well defined within treatment guidelines [4], [17], [19], [20], [21]. This retrospective analysis aimed to evaluate the long-term outcomes of SBRT for HCC performed at Erasmus University Medical Center (Erasmus MC). Our hypothesis was that SBRT for HCC had a durable effect on tumor control and could be delivered with limited toxicity thus making it an effective LRT to be considered amongst the other available options in the existing treatment paradigm.

## Materials and methods

For this retrospective analysis, the research protocol and patient consent form were approved by the institutional Medical Ethics Review Board (MEC 22–0695).

### Population

All patients diagnosed with HCC based on imaging and occasionally based on pathology were included in this study. All patients were treated with SBRT at Erasmus MC between January 1, 2008 and December 31, 2022. The eligibility criteria for SBRT were diagnosis of HCC, case review in a multidisciplinary liver board, BCLC stage 0, A or B and a non-cirrhotic liver or liver with cirrhosis Child-Pugh class A (CPA). The tumor size was limited to a maximum diameter of ≤ 6 cm and a maximum of three lesions with a cumulative diameter of ≤ 6 cm. SBRT was considered for patients who were not suitable for resection or thermal ablation. Patients suitable for TACE or with relapses after surgery, thermal ablation or TACE were considered possible candidates as well as patients awaiting transplantation. Exclusion criteria were uncontrolled portal hypertension, extrahepatic disease and active hepatitis infection.

### Treatment

The SBRT protocol for the treatment of HCC is based on the experience gained from our phase 1–2 trial of SBRT for liver tumors [22], [23]. This protocol, which has demonstrated promising results and minimal hepatoxicity in another phase 1–2 trial [12], involves a risk-adapted dose prescription where patients receive 6x8 Gy or 6x9 Gy, with one day interval between fractions and a maximum of three fractions per week.

Gross tumor volume (GTV) and organs at risk (OARs) definition was performed on the late arterial phase registered to the portal venous phase of a contrast enhanced CT-scan. Both scans were acquired in breath hold at the end of expiration. An MRI scan was acquired and registered to the arterial and venous CT phases to better depict GTV boundaries. No clinical target volume (CTV) margin was applied. A patient-specific planning target volume (PTV) margin was calculated based on the breathing amplitude and the distance of the target to the tracking surrogates (fiducial markers). Treatment planning and final dose calculation were performed using the previously mentioned late arterial phase contrast enhanced CT-scan and a Monte Carlo based dose calculation engine. Details of the used techniques are described in previous publications [17], [22], [24].

In case the 8 Gy per fraction treatment scheme was used, the primary target objective was to cover ≥ 95 % of the PTV volume with ≥ 48 Gy. A secondary target objective was to cover ≥ 95 % of the GTV volume with ≥ 54 Gy, while avoiding this dose level in the PTV margin. Underdosing of the PTV and GTV was allowed to spare the OARs. The prescription isodose could vary between 75 % and 80 % with a maximum dose in the target of 100 %. If a 9 Gy per fraction treatment scheme was used, the target dose objectives of the 8 Gy per fraction scheme were proportionally adjusted while the OAR constraints remained in place. An overview of target objectives and OAR constraints used in clinical practice at Erasmus MC for SBRT of HCC are available in Appendix A, [38], [39].

### Follow-up

Patient follow-up data up to June 30, 2023 were included in the analysis.

Medical information of the patients used in this study was collected retrospectively from the hospital’s electronic health record system HiX (ChipSoft, Amsterdam, The Netherlands) and the institutional TRENDY Trial database [17]. Patient and tumor baseline characteristics were composed from records created no more than three months prior to start treatment. For follow-up imaging, we selected MRI or CT images that were acquired every three months in the first year after SBRT treatment, every six months in the second and third year, and every 12 months thereafter, or best fitting this scheme. All baseline and follow-up images were re-evaluated for tumor size measurement and treatment response for the purpose of this study by an expert radiologist (F.E.J.A.W.) using the modified response evaluation criteria in solid tumors (mRECIST) [25].

Acute and late toxicity were evaluated using the common terminology criteria for adverse events (CTC AE) V4.03 [26].

### Endpoints and statistical analysis

The primary endpoint was Local (target) Control (LC). Secondary endpoints were, Time To (local or distant) Progression (TTP), Overall Survival (OS), Response Rate (RR), transplantation and toxicity. The baseline date was defined as the first day of SBRT treatment. For two patients who received a second SBRT treatment for a new occurring tumor, the date of start of the first radiotherapy treatment was taken as baseline.

LC probability was calculated from baseline to local progression. Patients were censored at the date of their last radiological evaluation or at the date of liver transplantation, if applicable.

TTP was defined as the time from baseline to progression (local or distant). Patients were censored at the date of liver transplantation if applicable or at the date of their last radiological evaluation, while a second analysis was performed ignoring liver transplantation.

OS was calculated from the first day of radiotherapy treatment. The Kaplan-Meier method was applied to analyze LC, TTP and OS.

RR was calculated using the best observed response defined as Complete Response (CR) or Partial Response (PR) measured on follow-up imaging.

The number of performed liver transplantations was investigated together with the time from baseline to transplantation. Transplantation surgery reports were evaluated for complications.

Toxicity was defined as an SBRT related ≥ grade 3 AE according to CTC terminology. Acute toxicity was evaluated on blood test outcomes and patients’ health records up to three months after the last radiotherapy treatment date while late toxicity was evaluated from patients’ health records starting three months after the last radiotherapy treatment date.

The delivered radiotherapy dose to targets and OARs was evaluated in MIM® 7.1.6 (MIM Software Inc. Cleveland, OH) using the DICOM files of the original treatment plans retrieved from the hospital picture archiving and communications system (PACS).

All collected and generated data were stored in Castor V2023.3.2.1 (Amsterdam, The Netherlands) electronic data capture (EDC) system where after it was exported to SPSS® V28.0.1.0 (IBM Corp., Armonk, NY) for evaluation and R Statistical Software V4.3.1 (R Foundation for Statistical Computing, Vienna, Austria) to perform statistical analysis.

## Results

### Study population

From January 1, 2008 to December 31, 2022, 52 patients were treated with SBRT for HCC at Erasmus MC. One patient objected to the use of their data and this patient was excluded. A total of 51 patients were enrolled in this study. Of this group, six patients previously participated in the institutional TRENDY Trial, a multicenter, prospective, randomized phase 2 trial comparing TACE using drug eluting beads (TACE-DEB) to SBRT in the treatment of HCC [17]. Nineteen patients had previously been treated for HCC lesions using surgery (9), RFA (19), TACE (3), alcohol ablation (1) or a combination of these techniques in case of recurrences or new occurring tumors over time. Six patients had non-target HCC lesions at the time of SBRT which were treated with RFA (3), TACE (1), SIRT (1) or wait and scan (1). Two patients received a second SBRT treatment (>2 years interval) for a new occurring tumor resulting in a total of 53 SBRT treatments for HCC. One patient did not continue treatment after 5 fractions. Patient baseline characteristics are presented in Table 1.

**Table 1:** Patient baseline characteristics.

Patient variable (n = 51*)	**Description**	**Number**	**%**
Age	Median [range]	71 [33–86]	
Sex	Male Female	43 8	84 16
ECOG performance status	Missing 0 1 2	15 20 13 3	− 56 36 8
Cirrhosis (CTP score)	No cirrhosis A5 A6	11 28 12	22 55 23
Cirrhosis etiology	HBV HCV Alcohol NASH	8 6 17 9	20 15 42.5 22.5
BCLC stage	0 A B C	8 37 5 1	16 72 10 2
Platelet counts (x10^9/L)†	Median [range]	158 [44–340]	
Portal hypertension‡	Yes No	30 21	59 41

* For two patients who received a second SBRT treatment for a new occurring tumor, the date of start of the first radiotherapy treatment was used to compose patient variables. † N=50 due to one patient missing data. ‡ Portal hypertension was evaluated in the records based on the presence of varices, splenomegaly or collaterals.

Abbreviations: ECOG = Eastern Cooperative Oncology Group; CTP = Child Turcotte Pugh; HBV = Hepatitis B virus; HCV = Hepatitis C virus; NASH = Nonalcoholic steatohepatitis, BCLC = Barcelona clinic liver cancer; HCC = Hepatocellular carcinoma.

The first three patients that received SBRT in this population were irradiated using the Siemens Primus Linear Accelerator (LINAC) (Siemens Oncology Systems, Concord, CA) and were positioned in the Elekta Stereotactic Body Frame (Elekta Oncology Systems, Stockholm, Sweden) with abdominal compression to minimize respiratory tumor motion. Since 2012, patients have been treated with the Cyberknife robotic stereotactic LINAC (Accuray, Sunnyvale, CA, US) with Synchrony® to track respiratory movement. Details of the applied techniques are described in previous publications [17], [22], [24]. The treatment characteristics of 53 SBRT treatments are presented in Table 2. Administered SBRT dose to targets and OAR, as per treatment planning, are available in Appendix B.

**Table 2:** Treatment characteristics.

Treatment variable (n = 53*)	**Description**	**Number**	
Number of HCC lesions per treatment	One Two Three	47 5 1	
Treated liver tumor†	No previous local treatment Local relapse after surgery Local relapse after RFA/MWA Local relapse after TACE	29 5 7 13	
Tumor diameter (mm)	Median [range]	26 [8–68]	
GTV volume (cc)	Median [range]	29 [1–169]	
PTV margin (mm)	5 6 7	16 28 9	
PTV volume (cc)	Median [range]	68 [6–355]	
Functional liver volume (cc)	Median [range]	1395 [885–3094]	
Fraction dose (Gy)	8 9	50 3	

* All 53 separate radiotherapy treatments were used to compose the treatment variables. † One patient received both RFA and TACE for the same tumor prior to SBRT.

Abbreviations: HCC = Hepatocellular carcinoma; RFA = radiofrequency ablation; MWA = microwave ablation; TACE = transarterial chemoembolization; GTV = gross tumor volume; PTV = planning target volume.

### Local control

**Fig. 1:**
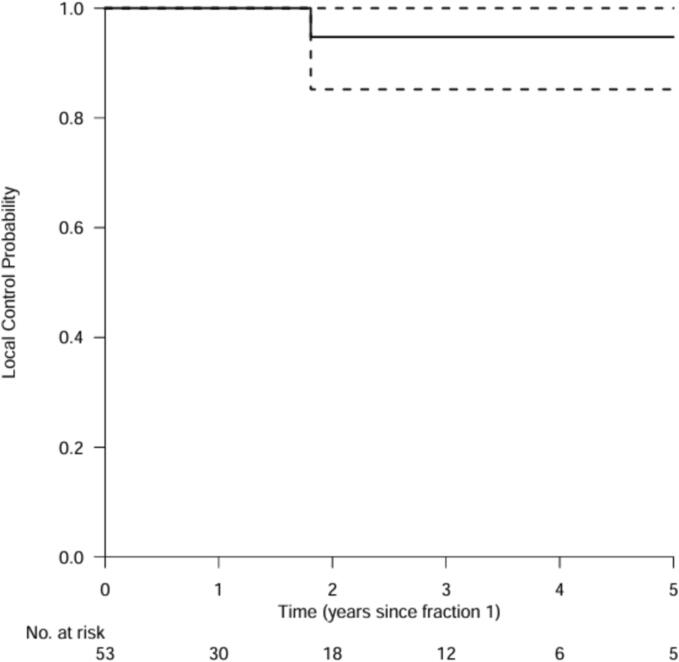
Local control defined as the time from baseline to local (target) progression. Patients were censored at the date of their last radiological evaluation or at the date of liver transplantation if applicable.

The median follow-up for radiological tumor response was 2.1 years [0.5–14.8]. The median local control was not reached. The analysis of LC for 53 SBRT treatments showed one event after 21 months of follow-up (Fig. 1). LC probability at 1, 2, 3 and 5 years were 100 %, 95 %, 95 % and 95 % respectively.

### Time to progression

**Fig. 2:**
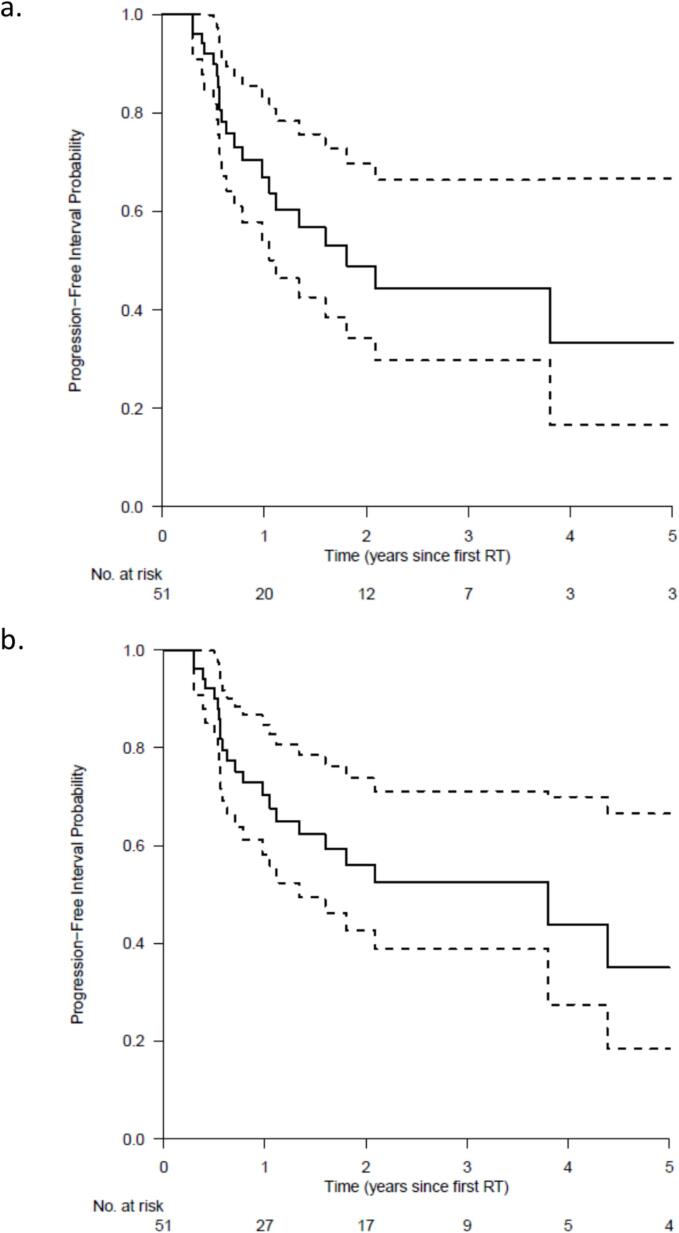
Time to progression defined as the time from first SBRT treatment to the time of first (local or distant) progression, (a) with censoring at liver transplantation and (b) without censoring at liver transplantation. Abbreviations: RT = radiotherapy treatment.

Median TTP (either local or distant) was 21.7 months [95 %CI, 13.3 to not reached]. The second analysis ignoring transplantation, resulted in a median TTP of 45.6 months [95 % CI, 16.1 to not reached] (Fig. 2).

A total of 24 patients had experienced disease progression during follow-up. The one patient with a local recurrence received systemic therapy to treat the progression of the target lesion as well as new occurring lesions in the liver. For the other 23 patients that experienced progressive disease at some point after SBRT, four patients had developed metastatic disease and 19 were diagnosed with new intrahepatic lesions of which four patients ultimately also developed metastatic disease. The intrahepatic disease progressions were treated with surgery (1), RFA (22), TACE (6), SBRT (2), SIRT (1), liver transplantation (3) or a combination of these techniques in case of recurrences or new occurring tumors over time. Extrahepatic progression was treated with surgery for occurring lung metastases (1) or systemic therapy (3) or best supportive care (3).

### Overall survival

**Fig. 3:**
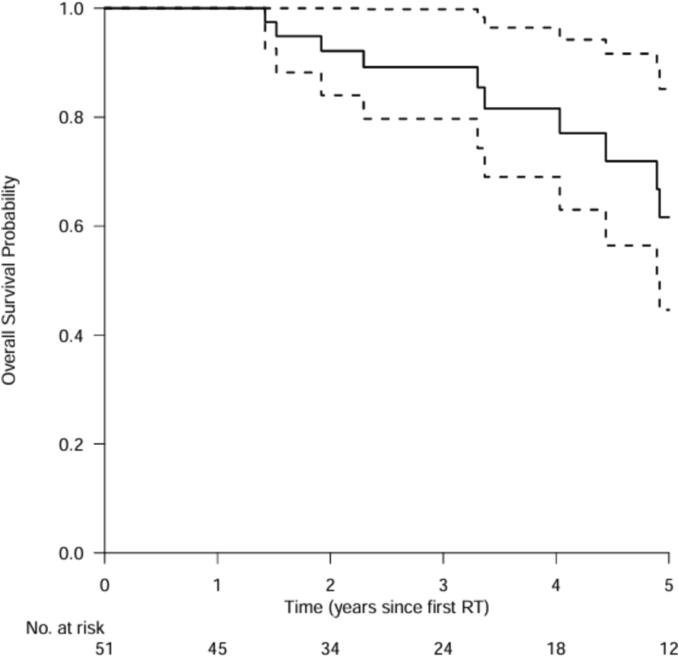
Overall survival. For the two patients that received a second SBRT treatment for a new occurring tumor, the date of start of the first radiotherapy treatment was taken as baseline. For patients alive, the date of follow-up was set at 30 June 2023. *Abbreviations: RT = radiotherapy treatment.*

The median follow-up for OS was 2.3 years [0.5–14.8]. Median OS was 7.1 years [95 % CI, 4.9 to not reached]. OS probability at 1, 2, 3 and 5 years was 100 %, 92 %, 89 % and 62 % respectively (Fig. 3).

### Response rate

The best observed RR (CR or PR) in this study was 96 % (see also Appendix C). Two patients (4 %) had stable disease (SD) as best observed response at three months radiological follow-up both undergoing liver transplantation before the next radiological follow-up. One patient did experience a local progression at 21 months but only after a previous PR.

### Transplantation

Thirteen patients underwent a liver transplantation after SBRT with a median time interval between baseline and transplantation of 7 months [range 3–16]. All transplantations were ultimately successful although four surgery reports stated that explantation was difficult or challenging due to post treatment fibrosis or adhesion caused by both SBRT as RFA.

### Toxicity

None of the patients in this study had any reported SBRT related CTC AE ≥ grade 3 acute or late toxicity. Two patients showed an asymptomatic deterioration in their CP score by two points at one month and five months follow-up respectively. One patient was presented with symptomatic anemia due to an episode of lower gastrointestinal bleeding possibly not related to SBRT at six months follow-up. This was classified as a grade 2 toxicity as it was effectively treated by temporarily stopping anticoagulant medication. The patient was known to have rectal angiodysplasia. There was no sign of bleeding in an upper gastrointestinal endoscopic exploration. The patient refused to undergo a colonoscopy. One patient experienced grade 1, mild gastrointestinal pain at 2 months of follow-up which was effectively treated by administering a proton pump inhibitor. The patient who did not continue treatment after 5 fractions suffered from increasing pain in the thorax; this was later identified as not-SBRT-related.

## Discussion

The outcomes of this study showed that SBRT resulted in excellent local control of HCC in a selected population, with only one tumor recurrence for 53 SBRT treatments, while there were no radiotherapy related severe toxicities identified. This confirms that SBRT has a durable effect on tumor control and can be delivered with limited toxicity making it an effective locoregional treatment for HCC that should be considered amongst the other available LRT options in the existing treatment paradigm.

The median LC in this study was not reached. LC probability at 1, 2, 3 and 5 years was 100 %, 95 %, 95 % and 95 % respectively, with only one reported tumor recurrence after 21 months of follow-up. This shows that SBRT can achieve an excellent local control in the treatment of HCC. The results are in line with comparable SBRT studies that reported 2- to 5-year LC rates of ≥ 90 % [11], [27], [28], [29]. SBRT appears favorable compared to the control probability of 50 % at 5 years for surgery and 70 % at 3 years for RFA reported by the American and European liver disease associations [3], [5]. The patient that did display a local recurrence after 21 months of follow-up suffered from a relative large tumor with a diameter of 68 mm and a volume of 355 cc, situated in a liver of 1450 cc functional volume with CPA cirrhosis. This patient received a six fractions of 8 Gy SBRT treatment covering 95 % of the PTV volume thereby adhering to the protocol. In other studies, larger tumors have been associated with poorer outcomes of SBRT and other LRT for HCC [30], [31].

The median TTP of 21.7 months and 45.6 months (censoring and ignoring liver transplant) in the current study is more favorable than the previously reported median TTP of 18.8 months and 18.8 months (censoring and ignoring liver transplantation) in the randomized multicenter TRENDY Trial. This difference may have been influenced by the longer follow-up period of the current study. Even though the median follow-up time is similar, 27.6 in this study versus 28.1 months in the TRENDY Trial, the current study included patients with a follow-up period of more than 10 years versus a maximum of 4 years in the TRENDY Trial. Other factors that might be of influence are a difference in patient characteristics such as percentage of patients suffering from cirrhosis (65 % vs. 83 %) and median tumor size (26 mm vs. 35 mm) for this study and TRENDY Trial respectively. Furthermore, the median age in this study was 71 years versus 62 years in the TRENDY Trial, where older patients might be more likely to die from other causes than disease progression. Our median TTP falls in the range of other reported SBRT outcomes with median values ranging from 6 to 47.8 months [11], [12], [32]. Studies reporting TTP after RFA, TACE, SIRT or a combination of ablative therapies report median TTP’s ranging from 12.8 to 28 months [33], [34], [35] though there is a large heterogeneity in characteristics of included patients between the compared groups. Overall, the data from this study shows a favorable TTP compared to reported data on alternative local ablative techniques.

The BCLC reports an expected median survival of > 5 years for stage 0 and stage A [20]. In this study, median OS was 7.1 years [95 % CI, 4.9 to not reached] with an OS probability at 1, 2, 3, and 5 years of 100 %, 92 %, 89 % and 61 % respectively. The large majority (88 %) of the population in this study was classified as BCLC 0 or A, which means that median OS after SBRT is at least comparable to the current predicted OS of the BCLC after surgery, transplantation or ablation for these stages. Other comparable studies report large variations of 5 years OS rates ranging from 24 % to 77.6 % [13], [15], [36]. This confirms that the results of this study fit well within the published literature, and that our population had a relatively good prognosis, giving the fact that OS in patients suffering from HCC is largely affected by the underlying liver disease [37].

SBRT for HCC at Erasmus MC is applied in a six-fraction treatment regimen using dose objectives and constraints that have proven their efficacy and safety before [12], [15]. Until now we have maintained a conservative approach in selecting patients eligible for SBRT of HCC resulting in a study population where 88 % of the patients fell within BCLC stage 0-A and liver cirrhosis was confined to CPA with 22 % of the patients having non-cirrhotic livers. No patients in this study had any reported SBRT related CTC AE ≥ grade 3 toxicity, however due to the retrospective nature of this study we acknowledge that we cannot completely rule out the risk of missing data. While comparable studies have reported limited toxicities, including CTC AE ≥ grade 3, they often consist of patients with less favorable characteristics than our population [11], [12], [13], [31]. These studies have also associated the risk of toxicity with tumor size and pretreatment hepatic function, possibly explaining the favorable toxicity outcome in our patient group.

This study witnessed a 96 % local response (CR and PR) rate after SBRT (Appendix C). It is notable that these results are in place even though 14 patients were treated with a lower dose coverage of the target than the desired 95 %. Of this group, 4 patients were even treated with a PTV coverage of less than 70 % (see also Appendix B). This indicates that a patient can still benefit from this treatment even if target coverage needs to be sacrificed to avoid OAR constraint violation. Best overall response (CR and PR) was calculated to be 92 % (Appendix C). Three patients experienced progressive disease despite having a good local response. HCC patients are often presented with multiple lesions (12 % in this study), recurrent lesions (55 % in this study) or likely to develop new lesions making the overall response rate results of an LRT such as SBRT dependent on the stage and course of the disease and the treatment approach [7].

The main limitation of this study is that it was a retrospective, non-randomized single-center study, based on a relatively small population. Furthermore, despite the maximum follow-up time of 14.8 years in this study, the majority of the patients (28/51) received SBRT in the last three years of the study period causing a relatively low follow-up for radiological tumor response and OS of 2.1 and 2.3 years respectively. Also, the population of this study could be considered heterogeneous involving patients from BCLC very early to more advanced stage HCC.

On itself this analysis does not hold the answer on how to implement SBRT in the HCC treatment regimen. However, in combination with other published studies, it may prove additional value. Moreover, the information in this publication can serve as a guideline for institutes planning to start an SBRT program for HCC.

SBRT for HCC shows promising results as an alternative treatment to other local ablative therapies. Its position within the treatment guidelines is however still not well defined. This analysis shows that radiotherapy for HCC can achieve excellent treatment outcomes in a well-defined group of patients, favorable to reported outcomes from other ablative therapies, and with no related CTC AE ≥ grade 3 toxicity. SBRT should be considered as one of the available local treatment options for HCC.

## CRediT authorship contribution statement

**Wilhelm den Toom:** Investigation, Methodology, Writing – original draft. **Eva M. Negenman:** Investigation, Methodology, Writing – review & editing. **Francois E.J.A. Willemssen:** Investigation, Writing – review & editing. **Erik van Werkhoven:** Formal analysis, Validation. **Robert J. Porte:** Writing – review & editing. **Roeland F. de Wilde:** Writing – review & editing. **Dave Sprengers:** Writing – review & editing. **Imogeen E. Antonisse:** Writing – review & editing. **Ben J.M. Heijmen:** Writing – review & editing. **Alejandra Méndez Romero:** Conceptualization, Methodology, Supervision, Writing – review & editing.

## Declaration of competing interest

The authors declare that they have no known competing financial interests or personal relationships that could have appeared to influence the work reported in this paper.
