# Supplementary material for: Long-term outcomes of more than a decade treating patients with stereotactic body radiation therapy for hepatocellular carcinoma
Source: Clin Transl Radiat Oncol. 2024 Oct 18;49:100878. doi: 10.1016/j.ctro.2024.100878 (PMC11541668; doi:10.1016/j.ctro.2024.100878)
Supplement: Supplementary Data 1 [file mmc1.docx]

| **Table A.1:** Target objectives and OAR constraints used in clinical practice for SBRT of HCC in a six-fraction treatment scheme. | |
| --- | --- |
| **Target** | **Objectives** |
| PTV coverage _(8Gy/fr)_ | 95% ≥ 48 Gy |
| PTV max dose | D_max_ ≤ D_PTV_ / 0.75 |
| GTV coverage _(8Gy/fr)_ | 95% ≥ 54 Gy |
| **OAR** | **Constraints** |
| Liver minus GTV(s) | D_mean_ ≤ 18 Gy* |
|  | > 800 ml < 23.4 Gy |
|  | NTCP ≤ 5%† |
| Stomach/ Small and large bowel | D_max_ ≤ 39 Gy,  V_30Gy_ ≤ 5cc |
| Esophagus | D_max_ ≤ 36 Gy |
| Heart | D_2cc_ ≤ 41Gy |
| Gallbladder | D_max_ ≤ 45Gy |
| Spinal cord | D_max_ ≤ 24 Gy |
| Kidney | D_2/3_ ≤ 19.2 Gy |
| * Currently liver minus GTV constraint Dmean ≤ 18 Gy (QUANTEC ) is used in the clinic where earlier the 22 Gy proposed by Dawson et al. was used (38). † The NTCP parameters are based on the Lyman model proposed by Dawson et al. (39).  *Abbreviations: PTV = planning target volume; GTV = gross tumor volume; OAR = organ at risk; NTCP = normal tissue complication probability.* | |
