# Supplementary material for: Long-term outcomes of more than a decade treating patients with stereotactic body radiation therapy for hepatocellular carcinoma
Source: Clin Transl Radiat Oncol. 2024 Oct 18;49:100878. doi: 10.1016/j.ctro.2024.100878 (PMC11541668; doi:10.1016/j.ctro.2024.100878)
Supplement: Supplementary Data 2 [file mmc2.docx]

| **Table A.2**: Administered radiation dose, as per treatment planning to targets and OARs (n=53). | | |
| --- | --- | --- |
| **Target** | **Objectives** | **Outcomes, median [range]** |
| PTV coverage (%) | 95% ≥ 48 Gy _(8Gy/fr n=51)_  95% ≥ 54 Gy _(9Gy/fr n=3)_ | 97.1% [54.9-99.4]  98.9% [98.5-99.1] |
| GTV coverage (%) | 95% ≥ 48 Gy _(8Gy/fr n=51)_  95% ≥ 54 Gy _(9Gy/fr n=3)_ | 100% [74.6-100]  100% [100-100] |
| **OAR** | **Constraints** |  |
| Liver minus GTV(s) | D_mean_ ≤ 22 Gy | 9.25 Gy [2.40-17.36] |
|  | > 800 ml < 23.4 Gy | 1195 cc [783-2840] |
|  | NTCP* ≤ 5% | 0% [0-9.22] |
| Stomach | D_max_ < 39 Gy  V_30Gy_ ≤ 5cc | 15.93 Gy [2.18-39.51]  0 cc [0-4.63] |
| Duodenum | D_max_ < 39 Gy  V_30Gy_ ≤ 5cc | 12.29 Gy [0.58-39.23]  0 cc (0-3.4] |
| Bowel | D_max_ < 39 Gy  V_30Gy_ ≤ 5cc | 14.41 Gy [0.43-44.59]  0 cc [0-5.05] |
| Esophagus | D_max_ ≤ 36 Gy | 8.82 Gy [0.13-37.83] |
| Heart | D_2cc_ ≤ 41Gy | 9.64 Gy [0.02-42.58] 2missing† |
| Gallbladder | D_max_ ≤ 45Gy | 19.61 Gy [0.28-51.13] n=41 |
| Spinal cord | D_max_ < 24 Gy | 4.96 Gy [0.77-20.17] |
| Kidney (Right) | D_2/3_ < 19.2 Gy | 1.47 Gy [0.07-12.46] |
| * The NTCP parameters are based on the Lyman model proposed by Dawson et al. (39). † In 2 cases the heart was not contoured as an OAR.  *Abbreviations: PTV = planning target volume; GTV = gross tumor volume; OAR = organ at risk; NTCP = normal tissue complication probability.* | | |
