# Supplementary material for: Long-term outcomes of more than a decade treating patients with stereotactic body radiation therapy for hepatocellular carcinoma
Source: Clin Transl Radiat Oncol. 2024 Oct 18;49:100878. doi: 10.1016/j.ctro.2024.100878 (PMC11541668; doi:10.1016/j.ctro.2024.100878)
Supplement: Supplementary Data 3 [file mmc3.docx]

| **Table A.3**: Best overall and best target response following mRECIST criteria. | | |
| --- | --- | --- |
|  | Treatments  53 |  |
| **Best overall response** |  |  |
| CR | 35 | 66% |
| PR | 12 | 23% |
| SD | 3 | 6% |
| PD | 3 | 6% |
| **Best target response** |  |  |
| CR | 44 | 83% |
| PR | 7 | 13% |
| SD | 2 | 4% |
| PD | 0 | 0% |
| * Some treatments did involve two or three lesions (Table 2), but they were evaluated as one.  *Abbreviations: CR = complete response; PR = partial response; SD = stable disease; PD = progressive disease.* | | |
